# *Synotis baoshanensis* (Asteraceae), a new species from Yunnan, China

**DOI:** 10.1186/1999-3110-54-17

**Published:** 2013-08-23

**Authors:** Ming Tang, Yu Hong, Qin-Er Yang

**Affiliations:** 1 Key Laboratory of Plant Resources Conservation and Sustainable Utilization, South China Botanical Garden, Chinese Academy of Sciences, Xingke Road, Tianhe District, 510650 Guangzhou, China; 2 University of Chinese Academy of Sciences, Beijing, 100049 China

**Keywords:** Asteraceae, Chromosome number, Floral microcharacter, Karyotype, Senecioneae, *Synotis baoshanensis*

## Abstract

**Background:**

*Synotis* (Asteraceae; Senecioneae) is a genus of about 55 species mostly distributed in the Sino-Himalayan region. During a botanical expedition in southwestern China in 2012, we found an unusual population of *Synotis* in southwestern Yunnan Province. To determine the taxonomic identity of the population, we carried out gross-morphological, floral micromorphological, and cytological observations.

**Results:**

Our gross-morphological observations have shown that the population is most similar to *Synotis auriculata* C. Jeffrey & Y. L. Chen in leaf shape and in the number of phyllaries and florets, but readily distinguishable by the glabrous stem and petiole (vs. glandular pubescent), the exauriculate petiole (vs. auriculate), the uppermost leaves obviously smaller than the middle ones (vs. almost equally sized), and the larger ray florets (4–4.5 mm vs. ca. 2.5 mm). The floral micromorphological observations on the population agree with previous reports for other species of *Synotis*. The chromosomes of the population are counted to be 2*n* = 40. Its karyotype is formulated as 2*n* = 20m + 14sm + 6st.

**Conclusions:**

The population was determined to represent a new species, i.e. *Synotis baoshanensis* M. Tang & Q. E. Yang, which is described herein. The species belongs to *Synotis* ser. *Microglossae*.

**Electronic supplementary material:**

The online version of this article (doi:10.1186/1999-3110-54-17) contains supplementary material, which is available to authorized users.

## Background

*Synotis* (C. B. Clarke) C. Jeffrey & Y. L. Chen (Asteraceae; Senecioneae) is a genus of about 55 species endemic to the Sino-Himalayan region except for *S*. *atractylidifolia* (Y. Ling) C. Jeffrey & Y. L. Chen, which occurs in northern China (Jeffrey and Chen 1984; Chen 1999; Nordenstam 2007; Chen et al. 2011; Tang et al. 2013). For China 44 species have been recorded in the genus (Chen et al. 2011; Tang et al. 2013).

During field work in southwestern China in February 2012, we discovered an unusual population of a species of *Synotis* in the Baihualing National Nature Reserve, Baoshan, southwestern Yunnan Province. The plants, which were at very late fruiting stage, appeared to be most similar to *S*. *auriculata* C. Jeffrey & Y. L. Chen in leaf shape and in the number of phyllaries, but different in the glabrous stem and petiole (vs. glandular pubescent), the exauriculate petiole (vs. auriculate), and the uppermost leaves obviously smaller than the middle ones (vs. almost equally sized). We revisited the site in October 2012 and successfully collected flowering specimens of the population in question. Upon closer examination we confirmed the differences mentioned above and found that the ray florets were larger than those of *S*. *auriculata* (4–4.5 mm vs. ca. 2.5 mm). We determined that the population represents a new species, i.e. *Synotis baoshanensis* M. Tang & Q. E. Yang, which is described herein.

## Methods

### Floral micromorphological character observations

To observe the anther collar and the anther endothecial cell wall thickenings of *Synotis baoshanensis* (voucher: *M. Tang* & *Y. Hong 322*, HAST, IBSC), we followed the methods described by Tang et al. (2013).

### Chromosomal observations

We studied the chromosomes of one population of *Synotis baoshanensis* from the type locality (voucher: *M. Tang* & *Y. Hong 322*, HAST, IBSC). Carbol fuchsin root tip squashes were made following Tang et al. (2013).

## Results and discussion

### Taxonomic treatment

**Figure 1:**
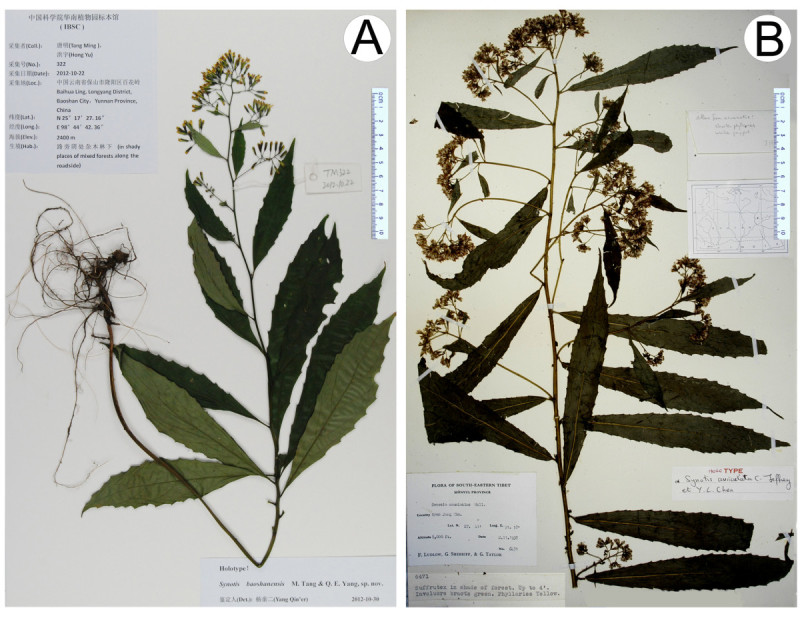
***Synotis baoshanensis*** **and** ***S.*** ***auriculata*** **. A**, *S*. *baoshanensis*, China, Yunnan, Baoshan City, Longyang District, Baihua Ling, *M*. *Tang* &*Y*. *Hong 322* (holotype, IBSC); **B**, *S*. *auriculata*, China, Xizang (Tibet), Nyam Jang Chu, *F*. *Ludlow*, *G*. *Sherriff* &*G*. *Taylor 6471* (holotype, BM).

**Figure 2:**
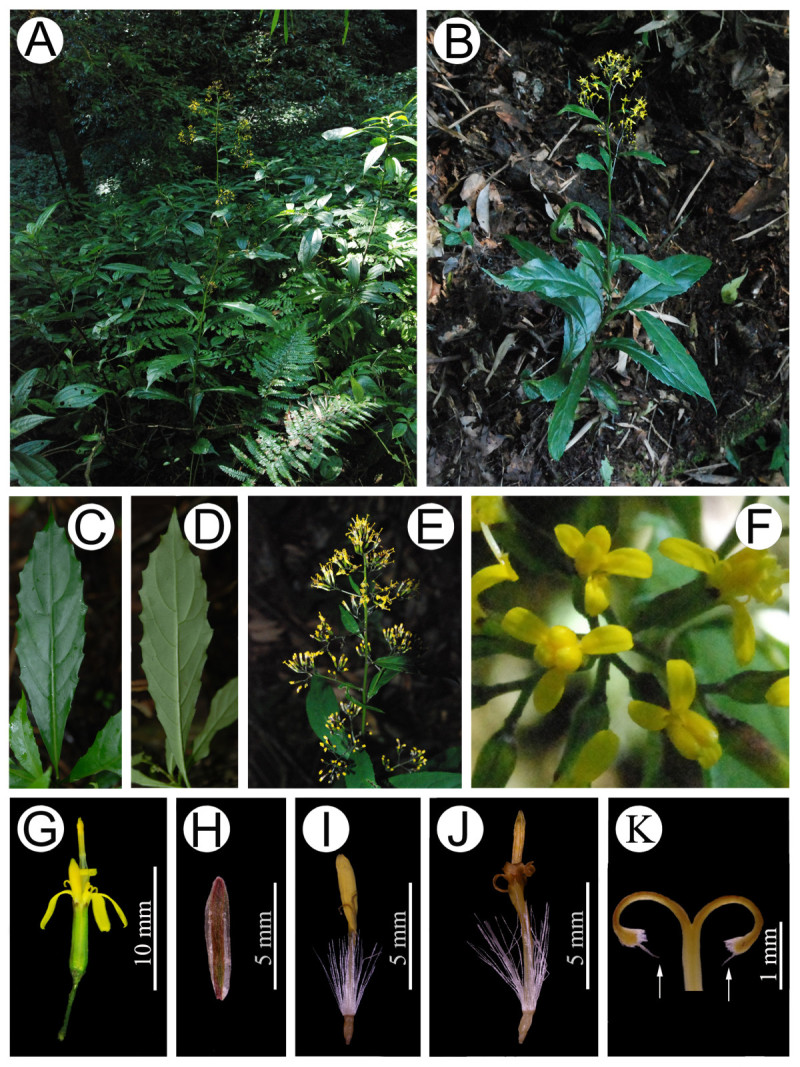
***Synotis baoshanensis*** **. A**, Habitat; **B**, Habit; **C**, Leaf blade (adaxial surface); **D**, Leaf blade (abaxial surface); **E**, Synflorescence; **F**, Capitula (top view); **G**, Capitulum (side view); **H**, Phyllary (abaxial surface); **I**, Ray floret; **J**, Disk floret; **K**, Style arms, note prominent central tuft of papillae on stigmas (arrows) which is much longer than the laterals. All from *M*. *Tang* &*Y*. *Hong 322* (HAST, IBSC) from the type locality.

***Synotis baoshanensis*** M. Tang & Q. E. Yang, sp. nov.—TYPE: CHINA. Yunnan, Baoshan City, Longyang District, Baihua Ling, alt. ca. 2,400 m a.s.l., in mixed forests, 22 Oct 2012, *M*. *Tang* & *Y*. *Hong 322* (holotype, IBSC; isotypes, HAST, IBSC). Figures 1A, and 2.

#### Description

Herbs, shrub-like, or subshrubs, 50–150 cm tall. Stems erect, branching, slender, glabrous. Leaves short-petiolate; petiole 1–1.5 cm long, glabrous, exauriculate; blade narrowly elliptic or oblong-elliptic, 5–22 cm long, 2–5 cm broad, base narrowly cuneate, margin prominently mucronate-serrate, apex acute-acuminate, papery, both surfaces glabrous, pinnately veined, lateral veins 12–18, arcuate-ascending, somewhat prominent abaxially; uppermost and branch leaves obviously smaller. Capitula radiate, numerous in lax axillary and terminal rounded compound corymbs; peduncles 2–3 mm long, densely pubescent, bracts and/or bracteoles 1 or 2. Involucres cylindrical, 5–5.5 mm long, 1.5–2 mm broad, calyculate; bracts of calyculus 2 or 3, ca. 1 mm long; phyllaries 5, oblong-linear, 5–5.5 mm long, 1–1.5 mm broad, margin scarious and subglabrous, apex obtuse and pubescent, often purplish. Ray florets 2 or 3; corolla ca. 8 mm long; tube ca. 4 mm long; lamina yellow, oblong, 3.5–4.5 mm long, ca. 1 mm broad, 3-denticulate, 2- or 3-veined. Disk florets 2 or 3; corolla yellow, 6–7 mm long, tube 3 mm long, infundibuliform limb exserted, narrow; lobes ovate-oblong, ca. 2 mm long, apex acute. Anthers ca. 3 mm long; anther tails 1/4 –1 3/4 as long as anther collar; appendages ovate; anther collars distinctly dilated at base. Style arms 1–1.2 mm long, fringed with short papillae, central tuft prominent, much longer than laterals. Achenes cylindrical, ca. 1.5 mm long, sparsely pubescent. Pappus white, ca. 5 mm long.

#### Etymology

The specific epithet ‘*baoshanensis*’ is derived from the type locality, Baoshan City, southwestern Yunnan Province, China.

#### Phenology

Flowering October–November; fruiting December.

#### Distribution and habitat

**Figure 3:**
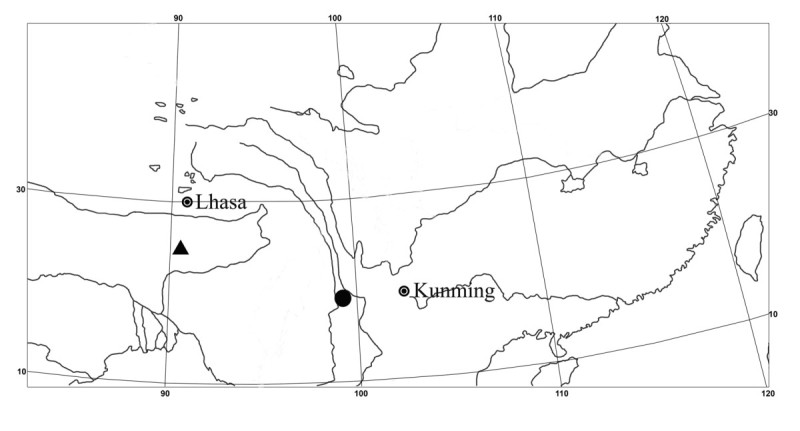
**Distribution of** ***Synotis baoshanensis*** **(●) and** ***S*** **.** ***auriculata*** **(▲).**

*Synotis baoshanensis* is currently known only from Baihua Ling, Longyang District, Baoshan City, southwestern Yunnan Province, China (Figure 3). It grows in shady, mixed forests between 2,350 and 2,550 m above sea level.

#### Floral micromorphological characters

**Figure 4:**
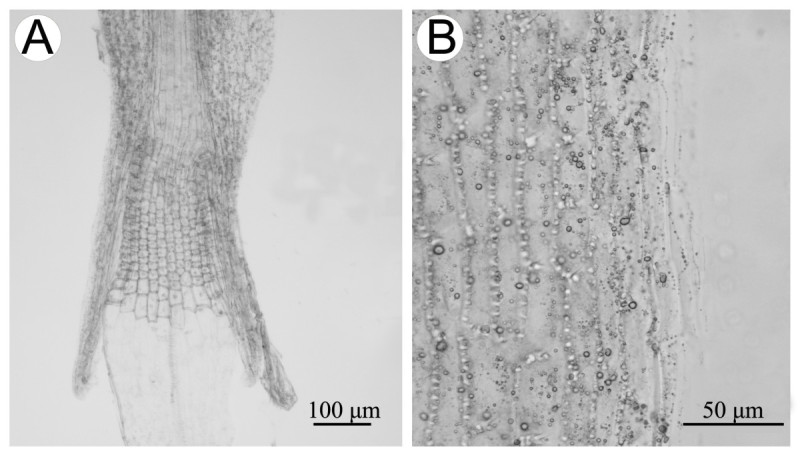
**Anther collar (A) and anther endothecial cell wall thickenings (B) of** ***Synotis baoshanensis*** **.** All from *M*. *Tang* &*Y*. *Hong 322* (HAST, IBSC) from the type locality.

As shown in Figure 4A, the anther collar of *Synotis baoshanensis* is balusterform, being basally dilated and consisting of larger cells, conforming to the results reported previously for some other species of *Synotis* (Jeffrey and Chen 1984; Tang et al. 2013). The anther endothecial cell wall thickenings were distributed along all the inner walls, and thus were radial (Figure 4B). The findings agree with previous reports for other species of *Synotis* (Jeffrey and Chen 1984; Tang et al. 2013). The placement of *Synotis* in subtribe Senecioninae as defined by Nordenstam (2007) is corroborated by the results.

#### Chromosome cytology

**Figure 5:**
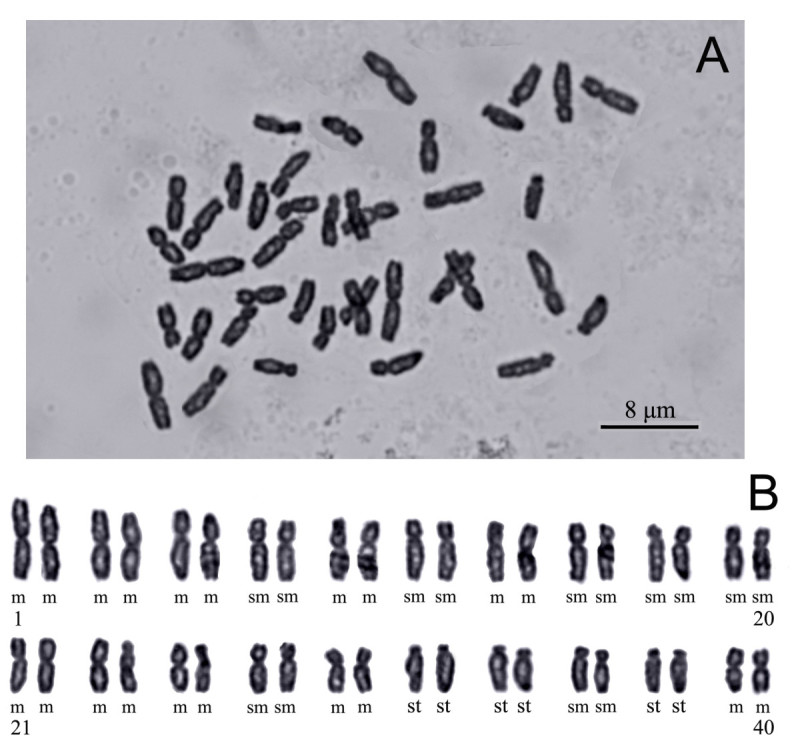
**Mitotic metaphase chromosomes (A) and karyotype (B) of** ***Synotis baoshanensis*** **.** All from *M*. *Tang* &*Y*. *Hong 322* (HAST, IBSC) from the type locality.

The metaphase chromosomes were determined to be 2*n* = 40 (Figure 5A). The karyotype analysis was performed on the basis of five well-spread metaphase cells. The chromosomes ranged in length from 4.0 to 6.7 μm, and total karyotype length was 107.0 μm. According to the chromosome nomenclature of Levan et al. (1964), *S*. *baoshanensis* had 20 median-centromeric (m), 14 submedian-centromeric (sm), and 6 subterminal-centromeric (st) chromosomes (Figure 5B), i.e. 2*n* = 40 = 20m + 14sm + 6st. The chromosomes showed a steady gradation in length from the longest to the shortest, with no evidence of bimodality.

As pointed out by Tang et al. (2013), *Synotis* is poorly known cytologically. In addition to the chromosome number of *S*. *baoshanensis* here counted, chromosome numbers for only six species have been reported (Mehra et al. 1965; Mehra and Remanandan 1975; Gupta and Gill 1981, 1989; Liu et al. 2006; Gupta et al. 2010; Tang et al. 2013; this study). *Synotis baoshanensis*, *S*. *changiana* Y. L. Chen and *S*. *xinningesis* M. Tang & Q. E. Yang are the only species in the genus for which the karyotype has been analyzed (Tang et al. 2013; this study). In chromosome number and chromosome morphology, the three species are very similar to each other, although in gross morphology *S*. *baoshanensis* is remarkably different from *S*. *xinningesis* and *S*. *changiana*, with *S*. *baoshanensis* belonging to ser. *Microglossae* C. Jeffrey & Y. L. Chen under sect. *Synotis* (see below); the latter two species belong to ser. *Synotis*, indicating that the *Synotis* may be karyologically rather constant.

#### Notes

**Figure 6:**
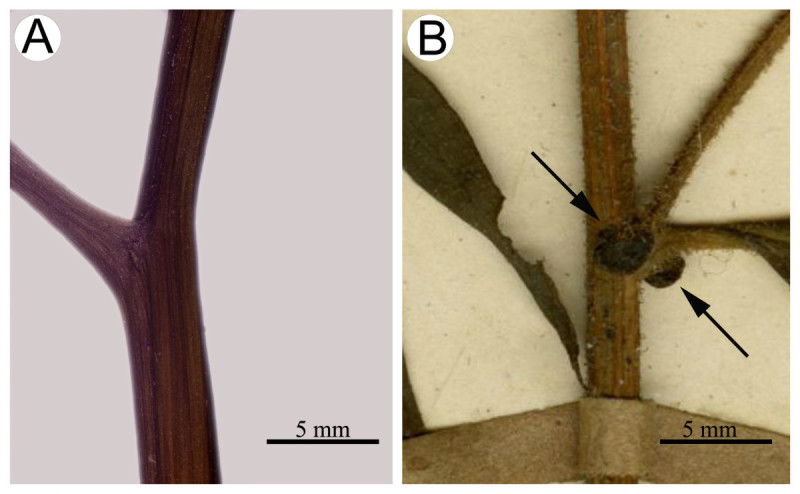
**Portion of stems of** ***Synotis baoshanensis*** **(A) and** ***S*** **.** ***auriculata*** **(B), showing detail of petiole bases and pubescence on stem and petiole.** A from *M*. *Tang* &*Y*. *Hong 322* (HAST, IBSC) from the type locality, note basally exauriculate petiole and glabrous stem and petiole; B from *F*. *Ludlow*, *G*. *Sherriff* &*G*. *Taylor* 6692 (E) from Nyam Jang Chu, Xizang (Tibet), China, note basally auriculate petiole (arrows) and glandular pubescent stem and petiole.

*Synotis baoshanensis* is most similar to *S*. *auriculata* (Figure 1B) in leaf shape and in the number of phyllaries and florets, but differs by the glabrous stem and petiole (vs. glandular pubescent) (Figure 6), the exauriculate petiole (vs. auriculate) (Figure 6), the uppermost leaves obviously smaller than the middle ones (vs. almost equally sized), and the larger ray florets (4–4.5 mm vs. ca. 2.5 mm).

As shown in Figure 3, *Synotis baoshanensis* is distributed in southwestern Yunnan, China, while *S*. *auriculata* is in southern Xizang (Tibet), China, so the two species are geographically isolated. Both species prefer similar habitats, growing in mixed forests at elevations between 2,000 and 2,600 m above sea level.

Five *Synotis* species closely related to each other and all endemic to Assam, India, i.e., *S*. *borii* (Raizada) R. Mathur, *S*. *jowaiensis* (Balak.) R. Mathur, *S*. *lushaensis* (C. E. C. Fisher) C. Jeffrey & Y. L. Chen, *S*. *rhabdos* (C. B. Clarke) C. Jeffrey & Y. L. Chen, *S*. *simonsii* (C. B. Clarke) C. Jeffrey & Y. L. Chen (Jeffrey and Chen 1984; Mathur, 1986), are somewhat similar to *S*. *baoshanensis* in habit, inflorescence shape, and pappus color, but all these Assam endemics are readily distinguishable from *S*. *baoshanensis* by, among others, the more or less broadly elliptic or oblong-elliptic leaves. In addition, *Synotis phupeakensis* H. Koyama, a species from northeastern Thailand (Koyama 1988) and also similar to *S*. *baoshanensis* in habit, inflorescence shape, and pappus color, is remarkably distinct by the large auricules at base of petiole, the campanulate involucres with 10 phyllaries, the ray florets 5, and the disk florets 7 or 8.

In leaf characters *Synotis baoshanensis* is somewhat reminiscent of *S*. *acuminata* (Wall. ex DC.) C. Jeffrey & Y. L. Chen, a species widely distributed in the eastern Himalayan region. Both species have narrowly elliptic or oblong-elliptic leaves, with the petiole not auriculate. However, *Synotis baoshanensis* is different from *S*. *acuminata* in the inflorescences round-topped (vs. flat-topped), the phyllaries 5 (vs. 3 or 4), the ray florets 2 or 3 (vs. 1) and oblong (vs. linear), and the pappus white (vs. stramineous). In fact, these two species should be classified under different series if following the infrageneric division proposed by Jeffrey and Chen (1984), and Chen (1999) (see below).

Jeffrey and Chen (1984), and Chen (1999) divided *Synotis* into two well-marked sections, sect. *Synotis* and sect. *Atractylidifoliae* C. Jeffrey & Y. L. Chen; all but one of the species (*S*. *atractylidifolia*) fall within the former, which itself is divisible into five not very clearly differentiated series. *Synotis baoshanensis* is characterized by leafy stems, round-topped inflorescences, white pappus, and 5 or 6-flowered, minutely radiate capitula, and thus can be readily referred to ser. *Microglossae* C. Jeffrey & Y. L. Chen, whereas Jeffrey and Chen (1984), and Chen (1999) have placed *S*. *acuminata* in ser. *Fulvipapposae* C. Jeffrey & Y. L. Chen, a series featuring, among others, flat-topped inflorescences and stramineous pappus. In China, ser. *Microglossae* now includes five species, namely *S*. *auriculata*, *S*. *baoshanensis*, *S*. *glomerata* (J. F. Jeffrey) C. Jeffrey & Y. L. Chen, *S*. *saluenensis* (Diels) C. Jeffrey & Y. L. Chen, and *S*. *triligulata* (Buch.-Ham. ex D. Don) C. Jeffrey & Y. L. Chen (Chen et al. 2011; this study), of which *S*. *baoshanensis* has the largest ray florets. The five species mentioned above can be distinguished by features in the following key.

### Key to *Synotis baoshanensis* and its related species in China

1. Corymbs dense, glomeruliform, 2–4 cm long *S*. *glomerata*.
2. Corymbs more lax, spreading, 4.5–10 cm long.
3. Capitula 4 mm long, 2.7 mm broad; phyllaries 8 *S*. *saluenensis*.
4. Capitula 3.5–4 mm long, 1.5 mm broad; phyllaries 4 or 5.
5. Leaves ovate or elliptic; ray florets 3 or 4 *S*. *triligulata*.
6. Leaves narrowly elliptic or narrowly oblong-elliptic; ray florets 2 or 3.
7. Base of petiole of cauline leaves auriculate; ray florets ca. 2.5 mm long *S*. *auriculata*.
8. Base of petiole of cauline leaves exauriculate; ray florets 4–4.5 mm long *S*. *baoshanensis*.

## Conclusions

*Synotis baoshanensis* is most similar to *S*. *auriculata* in leaf shape and in the number of phyllaries and florets, but differs by having the glabrous stem and petiole, the exauriculate petiole, the uppermost leaves obviously smaller than the middle ones, and the larger ray florets (4–4.5 mm). Both species belong to *Synotis* ser. *Microglossae*.

## Authors’ original submitted files for images

Below are the links to the authors’ original submitted files for images. Authors’ original file for figure 1 Authors’ original file for figure 2 Authors’ original file for figure 3 Authors’ original file for figure 4 Authors’ original file for figure 5 Authors’ original file for figure 6
