# Supplementary figures and images for: Synotis baoshanensis (Asteraceae), a new species from Yunnan, China
Source: Bot Stud. 2013 Aug 23;54:17. doi: 10.1186/1999-3110-54-17 (PMC5430305; doi:10.1186/1999-3110-54-17)

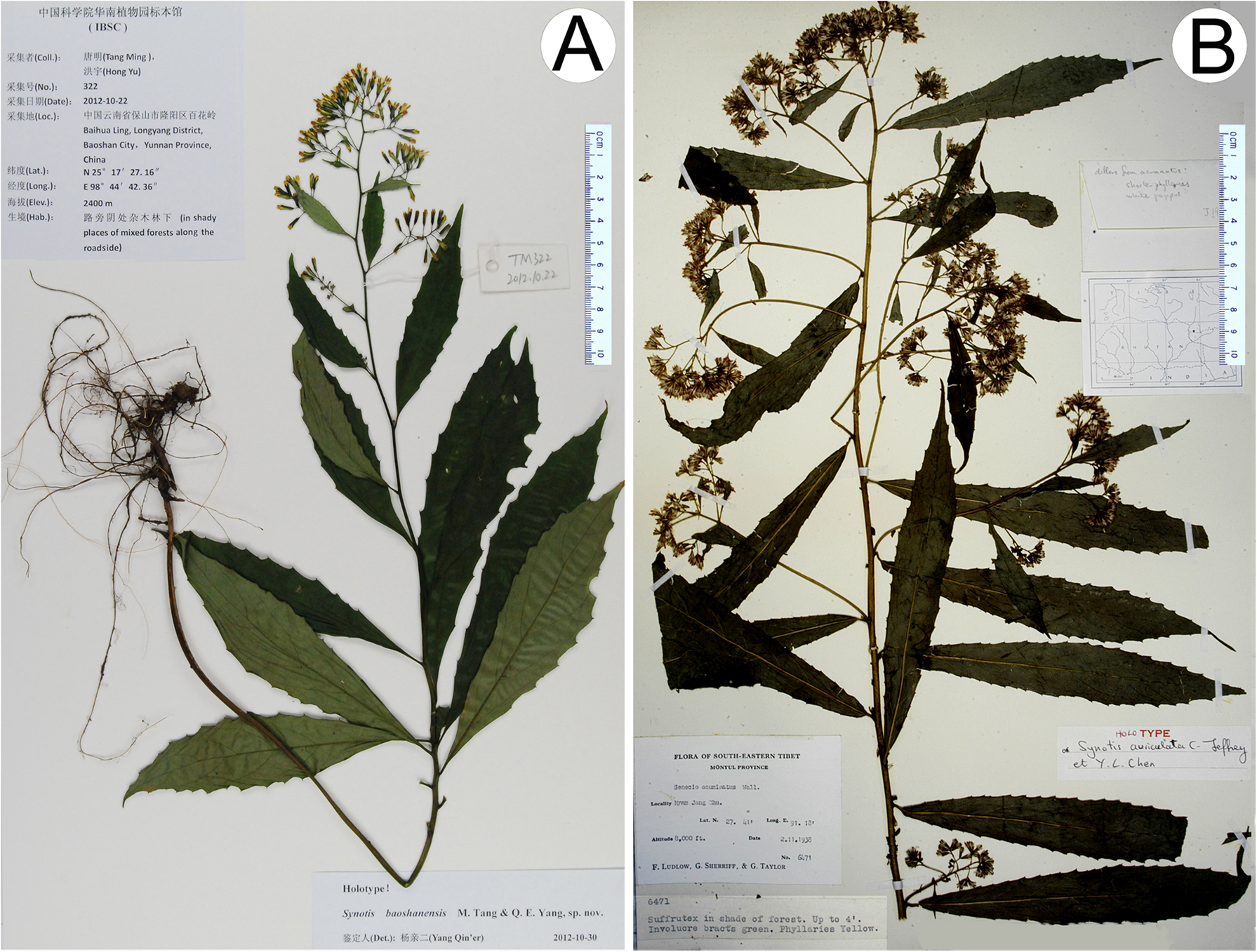

Supplement: Supplementary file 1 — Authors’ original file for figure 1 [file 40529_2012_14_MOESM1_ESM.tif]

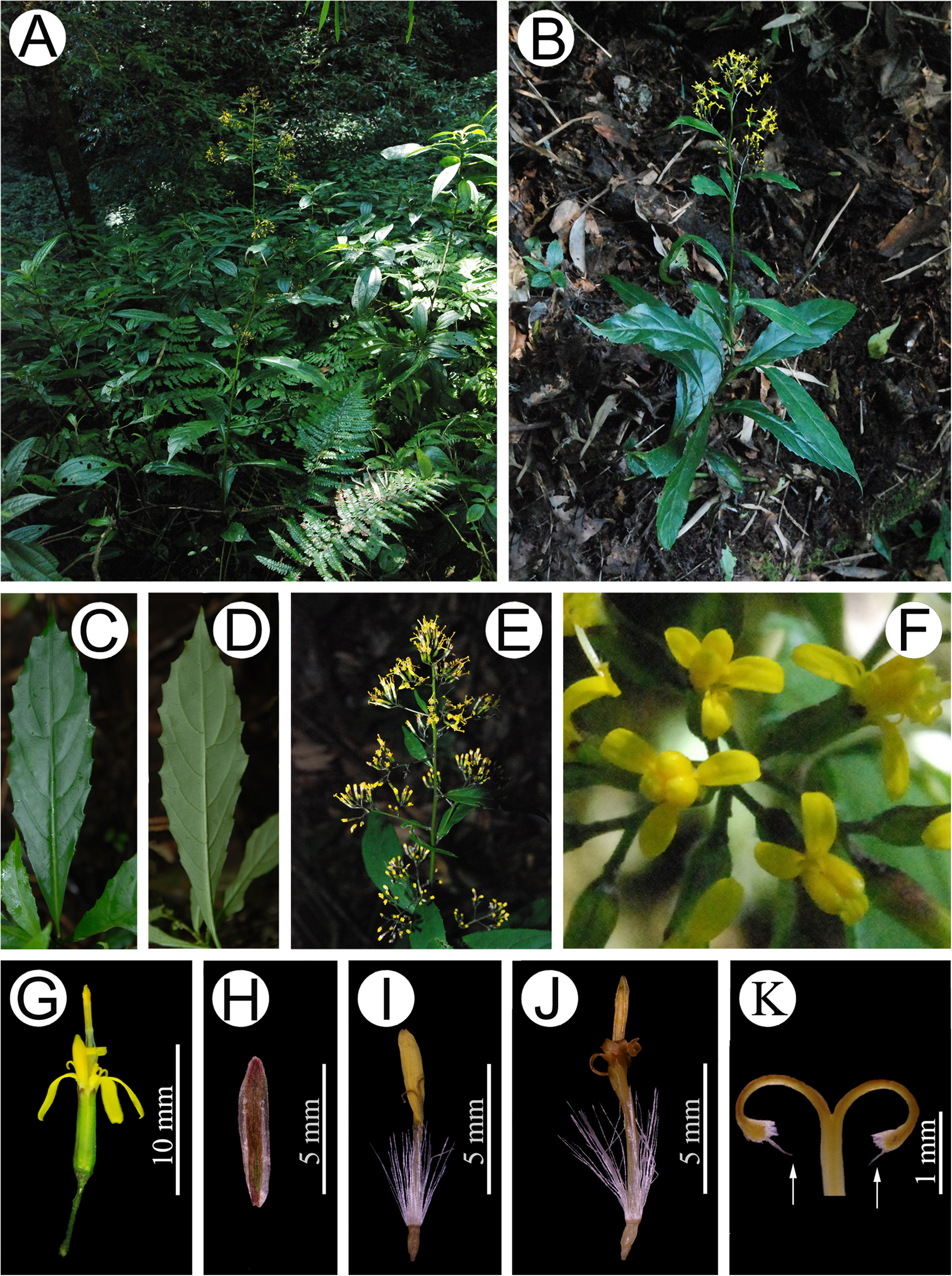

Supplement: Supplementary file 2 — Authors’ original file for figure 2 [file 40529_2012_14_MOESM2_ESM.tif]

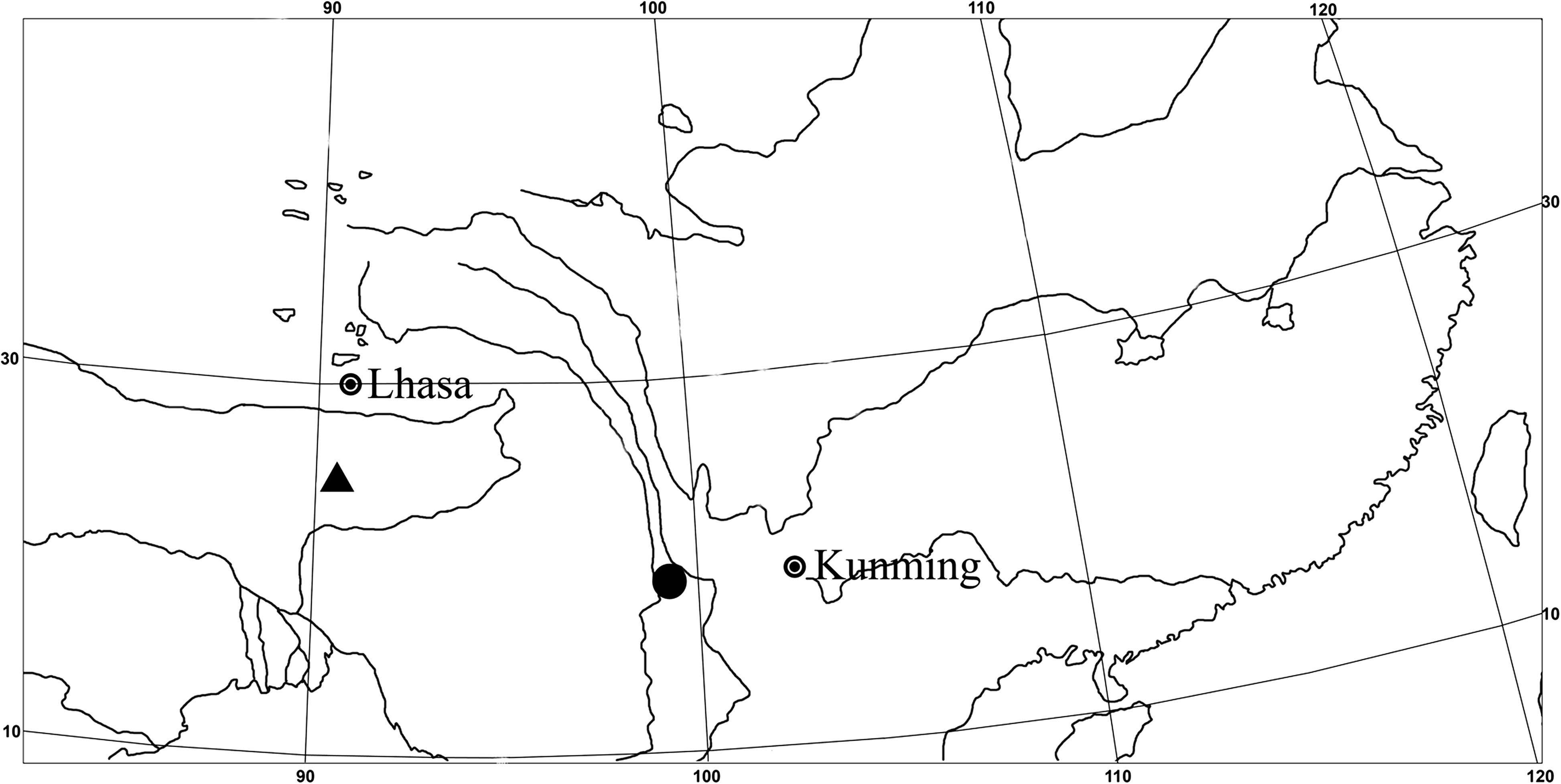

Supplement: Supplementary file 3 — Authors’ original file for figure 3 [file 40529_2012_14_MOESM3_ESM.tif]

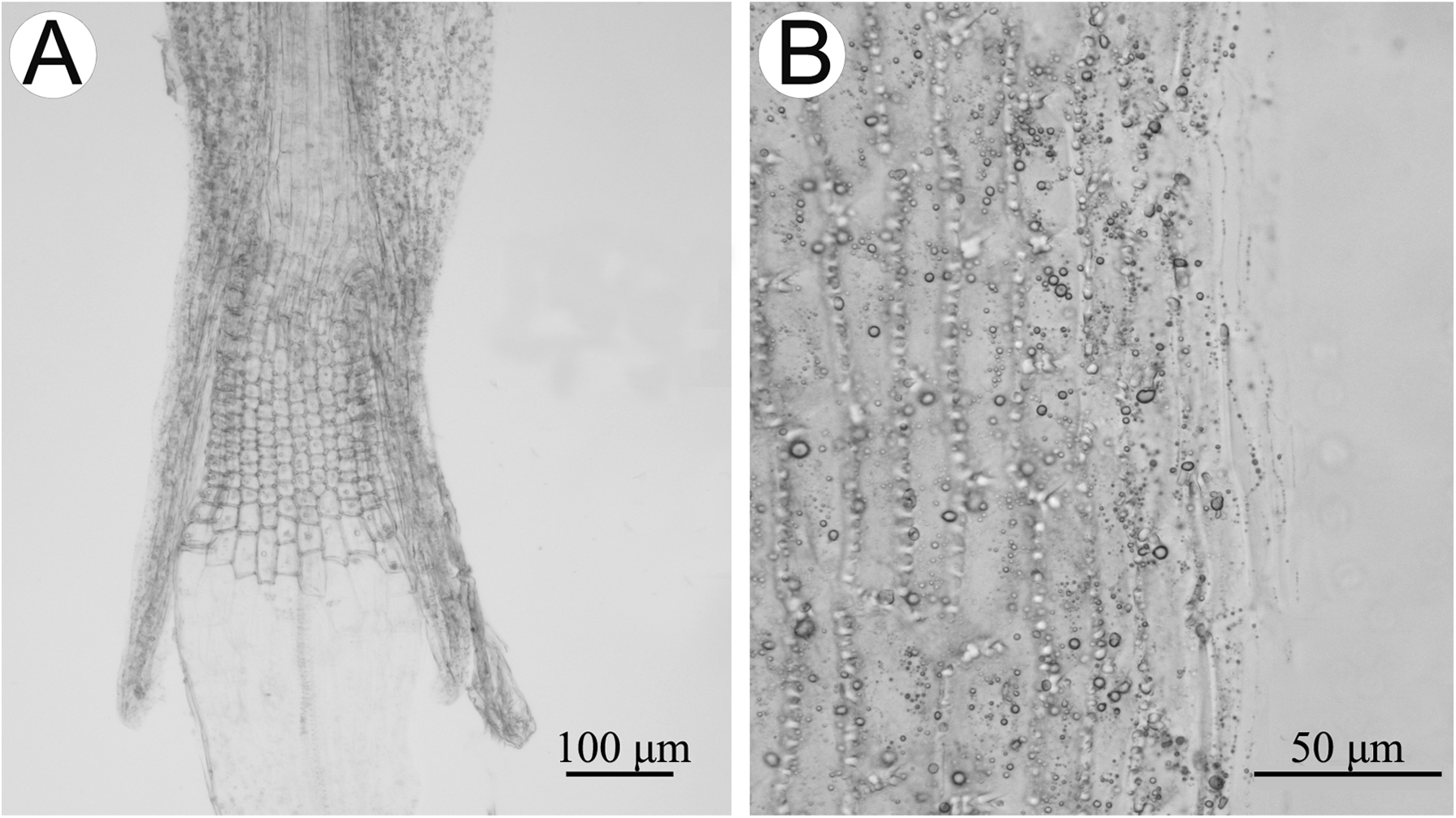

Supplement: Supplementary file 4 — Authors’ original file for figure 4 [file 40529_2012_14_MOESM4_ESM.tif]

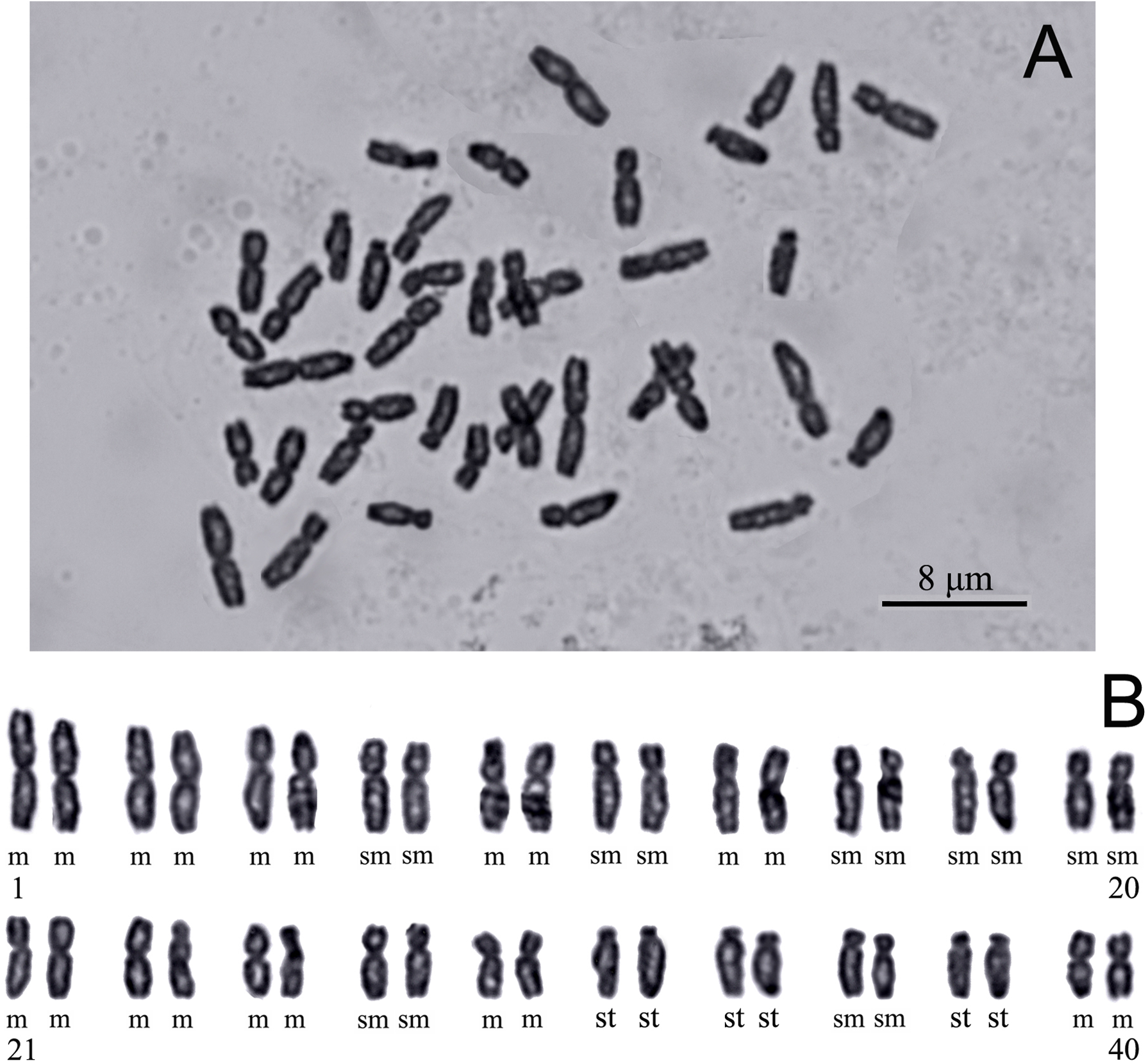

Supplement: Supplementary file 5 — Authors’ original file for figure 5 [file 40529_2012_14_MOESM5_ESM.tif]

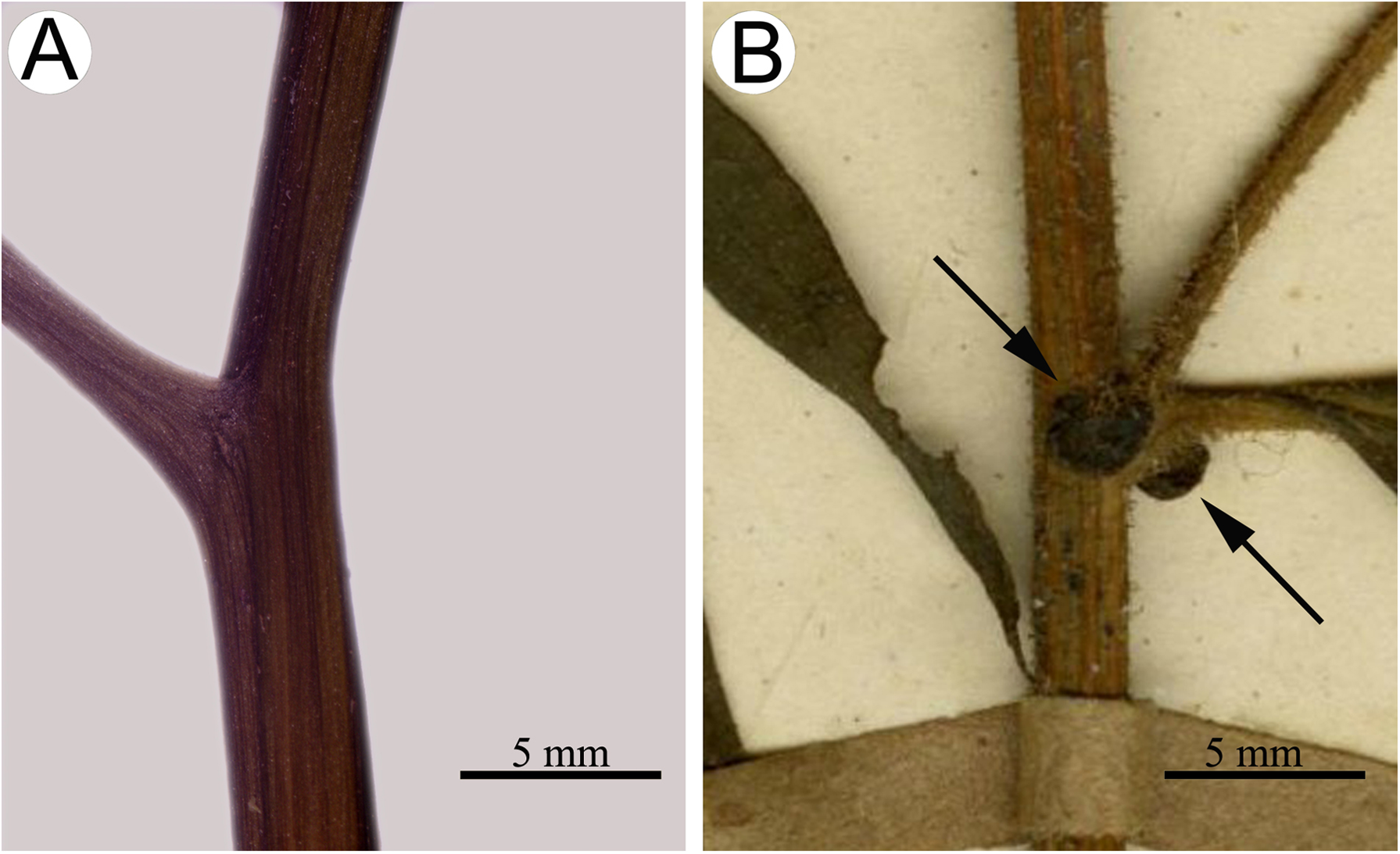

Supplement: Supplementary file 6 — Authors’ original file for figure 6 [file 40529_2012_14_MOESM6_ESM.tif]
